# Bacterial targets of fecal host miRNAs in high-fat diet-fed mice

**DOI:** 10.1371/journal.pone.0315871

**Published:** 2025-02-11

**Authors:** Laila Silamiķele, Ivars Silamiķelis, Patrīcija Paulīne Kotoviča, Jānis Kloviņš

**Affiliations:** Latvian Biomedical Research and Study Centre, Riga, Latvia; Defense Threat Reduction Agency, UNITED STATES OF AMERICA

## Abstract

The gut microbiome composition is intricately linked to the host’s health status, yet the mechanisms underlying its interaction with the host are not fully understood. MicroRNAs (miRNAs), facilitating intercellular communication, are found in bodily fluids, including the intestinal content, where they may affect the microbiome. However, their role in type 2 diabetes (T2D)-associated microbiome and treatment implications are not explored. Our study investigated how host miRNAs may influence gut microbiome changes related to metformin treatment in a T2D mouse model. Analyzing fecal and gut mucosal samples via small RNA sequencing, we correlated results with microbiome sequencing data, identifying miRNA-microbiome correlations, bacterial targets, and proteins targeted in these bacteria. Significant differences in miRNA expression based on diet and intestinal location were noted, with minor effects from metformin treatment in the proximal small intestine of non-diabetic male mice. Key fecal miRNAs targeting bacteria included mmu-miR-5119, mmu-miR-5126, mmu-miR-6538, and mmu-miR-2137, primarily affecting Oscillospiraceae_NOV, Lachnospiraceae_NOV, and Bacteroides. Our analysis of targeted proteins revealed diverse biological and molecular effects. Further research into miRNA-bacteria interactions could lead to new strategies for manipulating the gut microbiome in T2D and beyond.

## Introduction

The interaction between the host and its gut microbiome involving miRNAs has been described to be multidirectional [1]. While the gut microbiome influences host miRNA expression via microbiome-derived metabolites [2], the study by Liu and colleagues demonstrated for the first time that host-derived fecal miRNAs can specifically target bacterial genes, thereby modulating the gut microbiota [3]. Since then, several studies investigating the effects of host miRNAs on gut microbiota in different disease contexts have been performed, including colorectal cancer [4, 5], inflammatory bowel diseases [6, 7]⁠, insulin resistance [8]⁠, liver dysfunction [9], neurological disorders [10–12] as well as healthy mice and humans [13, 14].

MiRNAs, small non-coding RNAs involved in post-transcriptional gene regulation, exhibit robust stability relative to mRNAs [15]⁠. Extracellular miRNAs can be secreted and transported both via extracellular vesicles, such as microvesicles and exosomes, and in a form associated with high-density lipoproteins or Argonaute protein contributing to their extracellular stability [3]⁠⁠. Circulating miRNAs can be detected in body fluids, such as blood [16], saliva [17]⁠, cerebrospinal fluid [18], breast milk [19], and urine [20]⁠⁠, making them promising biomarkers. Fecal miRNAs have mostly been studied in the context of colorectal cancer [4, 5] and inflammatory bowel diseases [21, 22]. Despite their known functional role in the intestine, the involvement of fecal miRNAs in host-microbiome interaction is not yet fully understood [23].

Research of the gut microbiome and its impact on human health and disease has been a fast-growing area of biomedical science. Studies investigating host fecal miRNA effects on the microbiome in the context of microbiome-associated diseases have emerged only in recent years and have not yet been investigated in type 2 diabetes (T2D). T2D is a widespread metabolic disease, and strong evidence exists that the microbiome plays an important role in the pathogenesis of T2D and its interaction with therapy [24]⁠. The prevalence of T2D is constantly increasing–it has been estimated that 9.3% of adults aged 20–79 years (463 million adults) had diabetes (mostly T2D) globally in 2019, and it is expected to rise to 578 million by 2030 [25]⁠⁠. This global threat demands the optimization of therapeutic strategies and improvements in their efficiency. Metformin, a first-line therapy for T2D, exerts profound effects on the gut microbiome, yet its mechanisms of action remain incompletely elucidated [26]⁠. Additionally, the pleiotropic effects of metformin suggest potential interactions beyond its canonical targets.

Though the relationship between miRNA expression and microbiome-associated diseases and the association of gut microbiome dysbiosis to these diseases has been studied to some extent, studies exploring the communication between fecal miRNAs and the microbiome in the context of T2D are lacking. Furthermore, no research has yet investigated the associations between intestinal region-specific miRNAs and the local microbiome. Given the variable absorption of metformin across different regions of the gastrointestinal tract, with the proximal small intestine being the primary site [27], such studies could provide valuable insights into the variability of host miRNA-microbiome interactions. Therefore, the main objective of this study was to investigate whether metformin treatment-related changes in host miRNA expression correlate with alterations in the gut microbiome composition using a high-fat diet-induced T2D mouse model. We hypothesized that host miRNAs play a role in modulating the gut microbiome in response to metformin therapy and aimed to explore the influence of T2D background and sex on this interaction. Furthermore, we aimed to elucidate the potential functional targets of host miRNAs through bioinformatic analysis of targeted bacteria and their proteins. To our knowledge, this study represents the first investigation integrating small RNA-seq analysis with shotgun metagenomic sequencing data, providing novel insights into the interplay between host miRNAs and the gut microbiome in the context of T2D.

## Results

### MiRNA composition in feces

**Fig 1:**
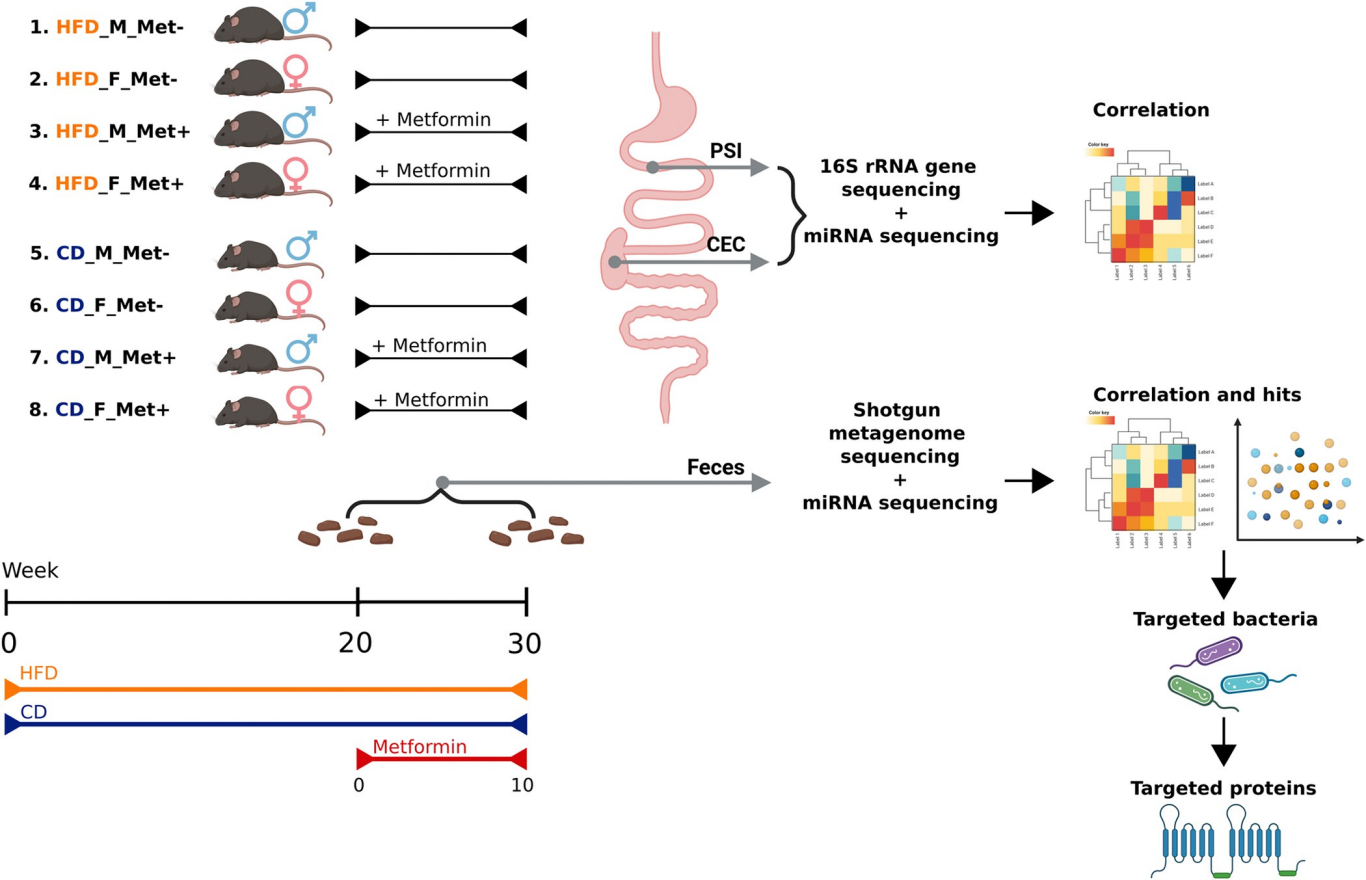
Experimental design of the study. (N = 24 cages with 3 animals each). Experimental groups: 1) HFD_M_Met-; 2) HFD_F_Met-; 3) HFD_M_Met+; 4) HFD_F_Met+; 5) CD_M_Met-; 6) CD_F_Met-; 7) CD_M_Met+; 8) CD_F_Met+. Abbreviations: HFD–high-fat diet; CD–control diet; M –male mice; F –female mice; Met- –mice not receiving metformin treatment; Met+ –mice receiving metformin treatment; PSI –proximal small intestine; CEC –cecum. After 20 weeks of HFD or CD feeding, ten weeks-long oral metformin treatment was provided for the corresponding experimental groups. Before and after the metformin treatment period (time points further denoted as 0 and 10, respectively), fecal samples were collected for the following sequencing analyses. At the endpoint of the study, gut mucosal (and luminal content only for 16S rRNA analysis) samples representing the proximal small intestine (the predominant site of metformin absorption) and cecum were collected for the following sequencing analyses.

A total of 48 fecal samples representing time points before and after ten weeks-long metformin treatment of 24 experimental units (cages with 3 animals) included in the animal experiment were used to determine the miRNA profile by small RNA-seq (Fig 1). These samples corresponded to the experimental units used for metagenome sequencing in fecal samples in a previous study using the same mice [28]. The median value of the obtained single-end reads was 2220516. After quality trimming, a median of 443840 reads was retained. The median percentage of annotated reads with miRBase (Mus musculus) was 0.61%. The median percentage of mapped reads, when mapped against the Mus musculus genome, was 69.53%.

**Fig 2:**
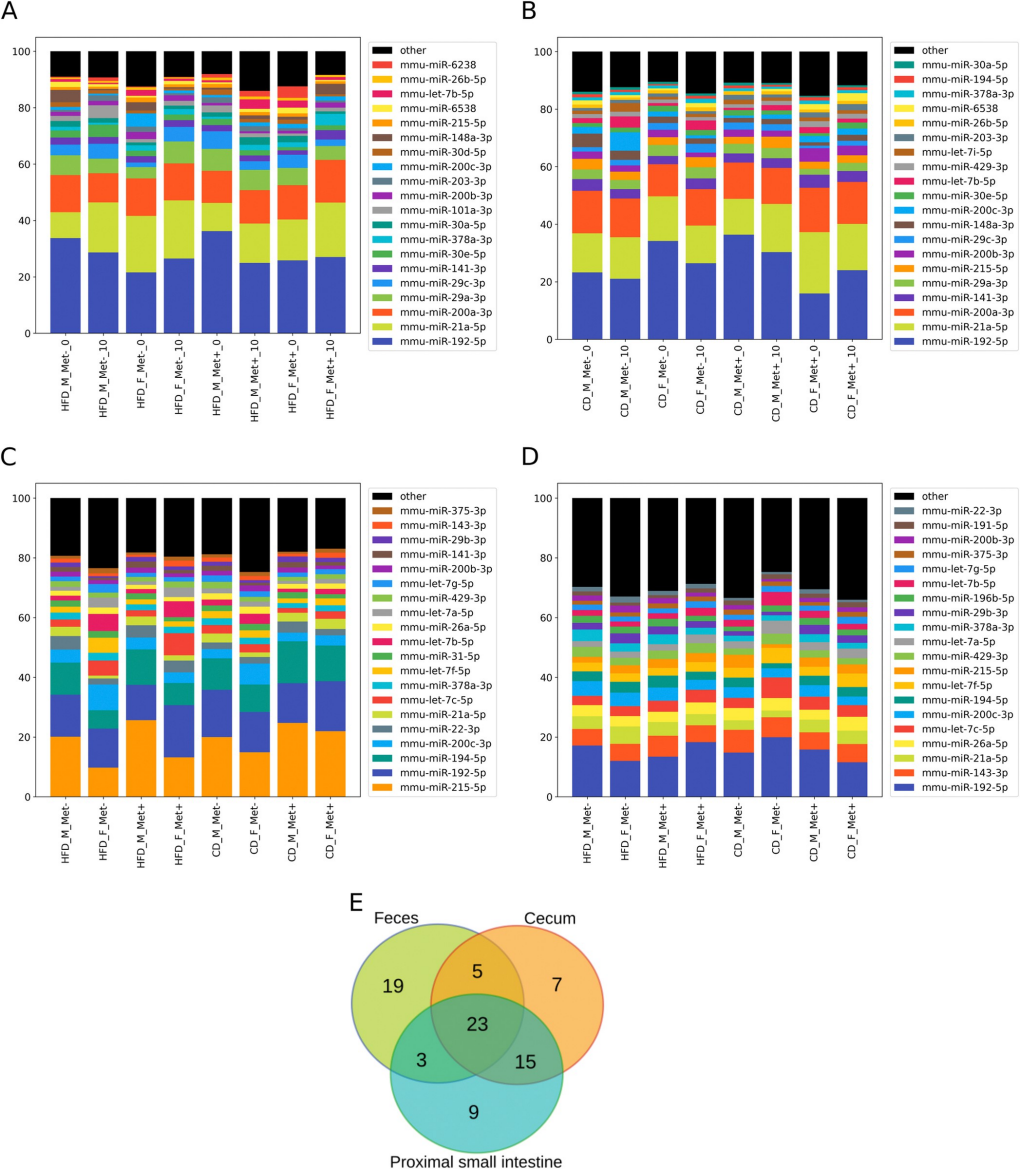
Mean percentage of the 20 most expressed miRNAs in each of the experimental groups. (A) Fecal miRNAs of high-fat diet-fed mice before (0) and after (10) metformin therapy. (B) Fecal miRNAs of control diet-fed mice before (0) and after (10) metformin therapy. (C) Mucosal miRNAs of the proximal small intestine at the time point after metformin therapy. (D) Mucosal miRNAs of the cecum at the time point after metformin therapy. (E) Venn diagram showing the counts of the common and the differentially expressed miRNAs between sample types (only top 50 miRNAs are included in the comparison). To align with the timing of mucosal sample collection, only fecal samples collected after metformin therapy were included in the comparison. N = 32 for fecal samples and N = 45 for gut mucosal samples.

After aligning reads to miRBase, 240 known mature miRNAs were detected by at least one read when samples were pooled together. Of the identified known miRNAs, 49 had only one read count in any of the samples, and 47 had at least 100 read counts. The top 20 miRNAs based on the read counts accounted for 81.7% of the total read counts. The elimination of substandard samples yielded 32 miRNA samples for further analyses. The expressions of the top 20 miRNAs in each of the experimental groups at the time points before and after the metformin treatment are shown in Fig 2A and 2B.

MiRNAs with the highest expression in fecal samples include mmu-miR-192-5p, mmu-miR-21a-5p, and mmu-miR-200a-3p followed by mmu-miR-29a-3p in HFD-fed mice and mmu-miR-141-3p in CD-fed mice. The expression of the top 20 miRNAs is relatively consistent between experimental groups.

### MiRNA composition in gut mucosa

In total, 48 samples representing the proximal small intestine and the cecum at the mucosal layer collected from 24 mice were sequenced by small RNA-seq (Fig 1). These samples corresponded to the experimental units used for microbiome determination with the 16S rRNA gene sequencing method in the respective intestinal samples in a previous study using the same mice [29]. The median value of the obtained single-end reads was 20698971. The median percentage of annotated reads with miRBase (Mus musculus) and against the Mus musculus genome was 0.83% and 42.73%, respectively. After aligning reads to miRBase, 296 known mature miRNAs were detected by at least one read when samples were pooled together in the proximal small intestine, while in the cecum 237 miRNAs were identified.

Of the identified known miRNAs in the proximal small intestine, 117 had less than 100 reads in all samples, and 179 had at least 100 reads in any of the samples. The top 20 miRNAs represented 81.6% of the total read counts. One sample was excluded from the further analysis due to low read count. In the cecum, 115 miRNAs had less than 100 reads in all samples, and 122 had at least 100 reads in any of the samples. The top 20 miRNAs comprised 69.3% of the read counts taken together. Two samples of this subset were excluded from future analysis as these had insufficient miRNA read counts (see S6 Table).

The relative expression of the top 20 miRNAs in each of the experimental groups in the mucosa of the proximal gut is shown in Fig 2C. In all groups, mmu-miR-215-5p prevailed, except for HFD_F_Met+ and HFD_F_Met-, where mmu-miR-192-5p, followed by mmu-miR-215-5p, dominated the miRNA composition. In all groups, mmu-miR-192-5p and mmu-miR-194-5p were the second and the third most abundant miRNA, respectively (except for HFD_F_Met-, where the third most abundant miRNA was mmu-miR-200c-3p). Overall, the miRNA composition of the proximal gut is relatively similar between experimental groups.

In the cecum, all groups were dominated by mmu-miR-192-5p, followed by mmu-miR-143-3p and mmu-miR-21a-5p in a different order (Fig 2D). In CD_F_Met- group, mmu-let-7c-5p, mmu-let-7b-5p, and mmu-let-7f-5p were among the most abundant miRNAs. The top 20 miRNAs showed a similar composition across groups, but they exhibited greater diversity compared to those in the proximal small intestine, as each miRNA accounted for a smaller percentage of the overall composition.

### Comparison of top 50 miRNAs between each of the sample types

When the top 50 miRNAs for each sample type were compared (Fig 2E), 23 miRNAs were found to be shared between all sample types. In turn, 15 miRNAs were shared between the proximal small intestine and cecum, while there were fewer miRNAs when comparing feces (collected after metformin treatment) and proximal small intestine or cecum samples– 3 and 6, respectively. Meanwhile, 18 miRNAs were highly expressed only in fecal samples compared to 9 unique miRNAs in the proximal small intestine and 6 miRNAs in the cecum.

### Differentially expressed miRNAs

**Fig 3:**
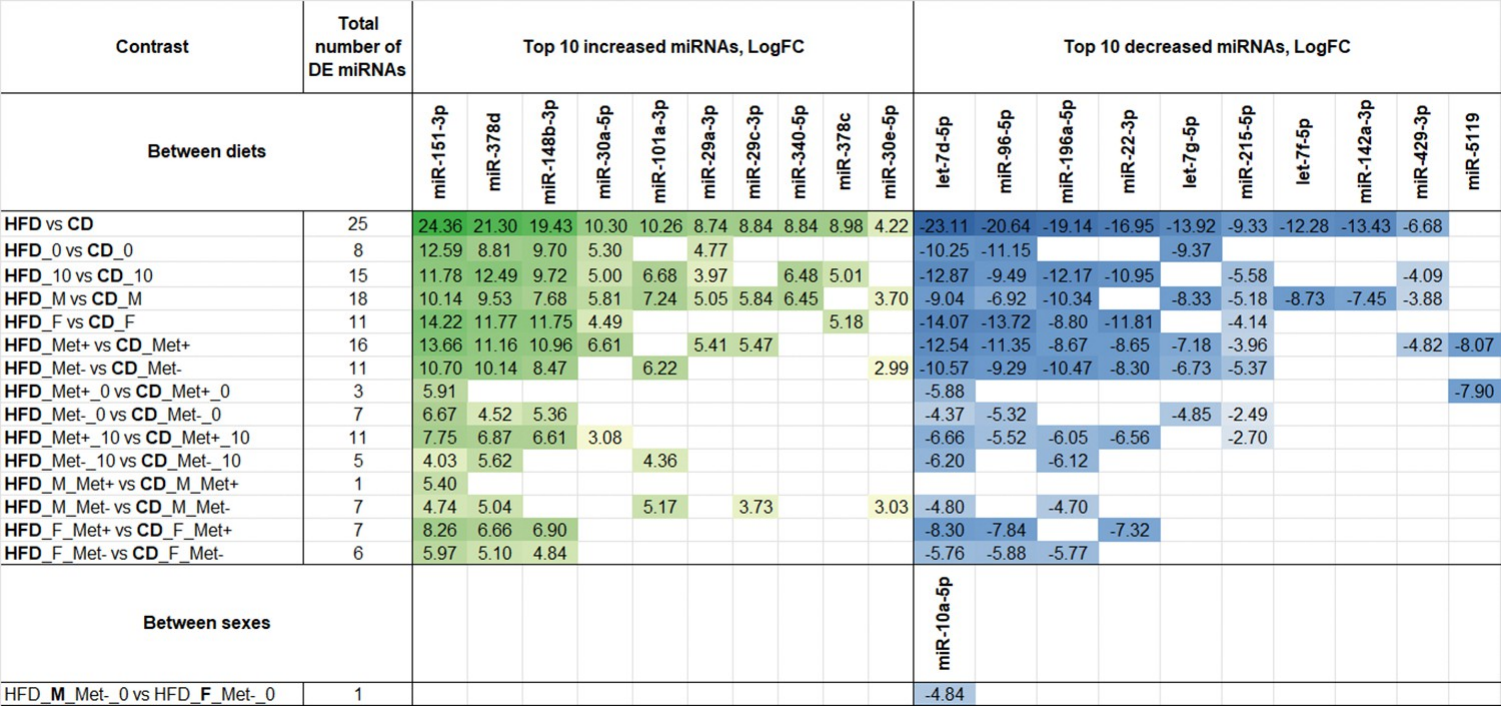
Summary of differentially expressed miRNAs in fecal samples using different contrasts. The top 10 significantly differentially expressed (DE) (FDR < 0.05) miRNAs are shown for each comparison, N = 32. Abbreviations: HFD–high-fat diet; CD–control diet; M–male mice; F–female mice; Met-–mice not receiving metformin treatment; Met+–mice receiving metformin treatment; 0 –time point before metformin treatment; 10 –time point after ten weeks long metformin treatment.

**Fig 4:**
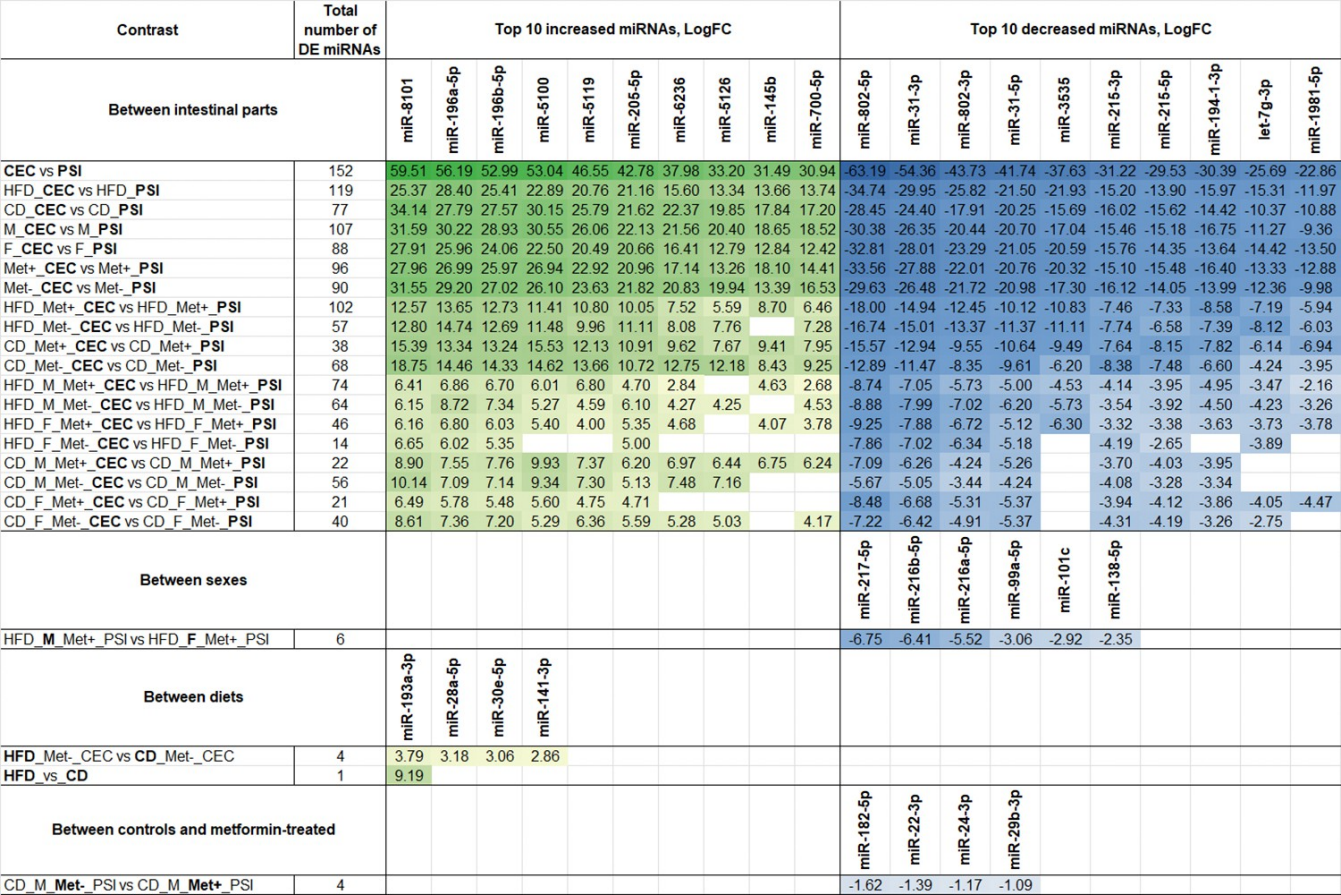
Summary of differentially expressed miRNAs in gut mucosal samples representing proximal small intestine and cecum using different contrasts. The top 10 significantly differentially expressed (DE) (FDR < 0.05) miRNAs are shown for each comparison, N = 45. Abbreviations: HFD–high-fat diet; CD–control diet; M–male mice; F–female mice; Met-–mice not receiving metformin treatment; Met+–mice receiving metformin treatment; PSI–proximal small intestine; CEC–cecum. Differential expression analysis of fecal samples revealed that the most pronounced differences were observed between HFD-fed and CD-fed mice at both time points before and after metformin treatment. The mmu-miR-151-3p showed the highest increase in the HFD group compared to the CD group, appearing consistently across all diet comparisons with the highest LogFC of 24.36. Conversely, mmu-let-7d-5p was significantly reduced in the HFD-fed mice in almost every contrast, with its lowest LogFC at -23.11. FDR was < 0.001 in both cases. In addition, in HFD-fed mice not receiving metformin treatment at the time point before the initiation of the treatment, a significant sex-related difference was detected for mmu-miR-10a-5p (LogFC = -4.84, FDR = 0.009). No effect of metformin treatment or any longitudinal differences between any of the analyzed contrasts were observed.

We compared the relative expression of different miRNAs across experimental groups in fecal and gut mucosal samples representing the proximal small intestine and cecum. This analysis was based on contrasts formed by combinations of the following factors: metformin use, time point (for fecal samples), intestinal site (for gut mucosal samples), diet type, and sex. Contrasts with significant differences for fecal sample analysis and differentially expressed miRNAs are shown in Fig 3 and S1 Table. Contrasts with significant differences for intestinal sample analysis and differentially expressed miRNAs are shown in Fig 4 and S2 Table.

As for gut mucosal samples, only cross-sectional contrasts were feasible due to the collection of the samples at a single time point–upon the termination of the experiment. Main comparisons aimed to investigate the differences between intestinal parts, sex, diet type, and the effect of metformin treatment, similar to the analysis done with fecal samples. The most pronounced differences were found between both studied intestinal parts. Some differences were detected depending on metformin treatment status, sex, and diet type. The highest number of differentially expressed miRNAs was observed between intestinal parts in HFD-fed mice. In addition, more pronounced changes were observed in all male experimental groups, characterized by a higher number of differentially expressed miRNAs and greater LogFC values, when each of the sexes was contrasted separately.

The top miRNAs with increased expression in the cecum compared to the proximal small intestine include mmu-miR-8101, mmu-miR-196a-5p, mmu-miR-196b-5p, mmu-miR-5100, mmu-miR-5119, and mmu-miR-205-5p. In turn, top depleted miRNAs in cecum samples, when compared to the proximal small intestine, include mmu-miR-802-5p, mmu-miR-802-3p, mmu-miR-31-3p, mmu-miR-31-5p, mmu-miR-3535, mmu-miR-215-5p, and mmu-miR-215-3p. Sex-specific differences emerged in the proximal small intestine among HFD-fed mice receiving metformin treatment, with male mice showing a significant decrease in the expression of six miRNAs including mmu-miR-217-5p, mmu-miR-216a-5p, mmu-miR-216b-5p, mmu-miR-99a-5p, mmu-miR-101c, and mmu-miR-138-5p. A significant diet effect was detected only in the cecum of mice not receiving metformin, where four miRNAs were increased in HFD-fed mice. When comparing diets across all samples, mmu-miR-193a-3p was found to be increased in HFD-fed mice (LogFC = 9.19, FDR = 0.03). Metformin effect was found only in the proximal small intestine of CD-fed male mice, where mmu-miR-22-3p, mmu-miR-29b-3p, mmu-miR-24-3p, and mmu-miR-182-5p were decreased in mice not receiving treatment compared to the treated ones.

### MiRNA correlation with gut microbiome

#### Correlation of fecal miRNAs with the fecal microbiome

We performed a correlation analysis between miRNAs and previously identified gut microbiome at the genus level independent of metformin treatment status using the sequencing data obtained from fecal samples. Due to the significant differences in gut microbiome composition previously reported in a study using the same mice [28], including a decreased relative abundance of Bacteroides and an increased relative abundance of Lactobacillus and Parabacteroides in HFD-fed mice compared to CD-fed mice, along with the miRNA expression differences observed in the current analysis, correlation analysis was performed separately for each dietary subgroup.

**Fig 5:**
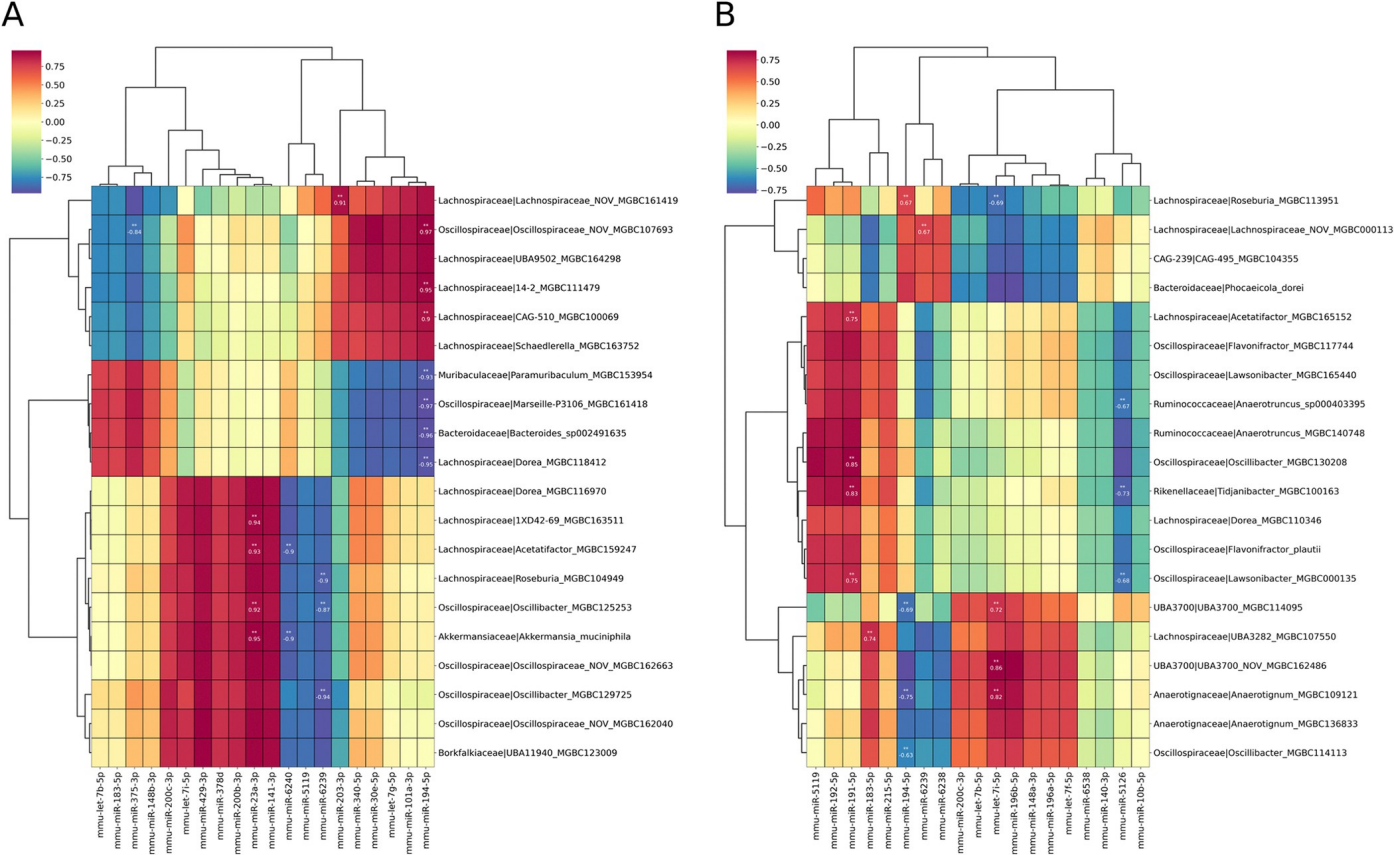
Clustered image map indicating Pearson correlation through sPLS latent components between fecal miRNAs and gut microbiome representatives identified by small RNA-seq and shotgun metagenomic sequencing, respectively. (A) Analysis of high-fat diet-fed mice, N = 6 (intersection of miRNA and metagenome datasets). (B) Analysis of control diet-fed mice, N = 11 (intersection of miRNA and metagenome datasets). The color key indicates the correlation levels, with blue and red color denoting negative and positive correlation, respectively. Only the top 20 strongest representatives from each dataset have been included in the plot. The heatmap is annotated with statistically significant correlation values, marked with * for FDR < 0.05 and ** for FDR < 0.001.

Analysis in HFD-fed mice (Fig 5A) revealed a very strong positive correlation between miR-8 family members (mmu-miR-200b-3p, mmu-miR-200c-3p, mmu-miR-141-3p, and mmu-miR-429-3p), mmu-miR-378d, mmu-let-7i-5p, and mmu-miR-23a-3p, and the abundance of Akkermansia muciniphila (r up to 0.95, FDR < 0.001), and Lachnospiraceae members (Acetatifactor, Roseburia, Dorea, and 1XD42-69), Borkfalkiaceae representative UBA11940, and Oscillospiraceae members (Oscillibacter and Oscillospiraceae_NOV) (r up to 0.94, FDR < 0.001). Likewise, the abundance of these genera was very strongly negatively correlated with another set of miRNAs containing mmu-miR-5119, mmu-miR-6239, and mmu-miR-6240 (r up to -0.94, FDR < 0.001), and to a lesser extent with mmu-miR-203-3p.

A set of miRNAs, including mmu-miR-101a-3p, mmu-miR-194-5p, mmu-miR-30e-5p, mmu-let-7g-5p, and mmu-miR-340-5p showed a strong negative correlation with Bacteroides, Paramuribaculum, Dorea, and Marseille-P3106 (r up to -0.97, FDR < 0.001). These miRNAs were, in turn, positively correlated with Oscillospiraceae_NOV (r = 0.97, FDR < 0.001) and Lachnospiraceae representatives (r up to 0.95, FDR < 0.001), including Schaedlerella and 14–2, among others. In contrast, a different set of miRNAs, including mmu-miR-375-3p, mmu-miR-148b-3p, mmu-let-7b-5p, and mmu-miR-183-5p, displayed a negative pattern of correlation (r up to -0.84, FDR < 0.001) with the aforementioned bacterial genera.

In CD-fed mice (Fig 5B), a miRNA set including let-7 family members (mmu-let-7b-5p, mmu-let-7i-5p, and mmu-let-7f-5p), mmu-miR-200c-3p, mmu-miR-148a-3p, and miR-196 representatives (mmu-miR-196b-5p and mmu-miR-196a-5p) showed the strongest positive correlation with members of Borkfalkiaceae (UBA3700_NOV) and Anaerotignum (r up to 0.86, FDR < 0.001). Conversely, these miRNAs negatively correlated with Roseburia (r = -0.69, FDR < 0.001), Phocaeicola dorei, and Alphaproteobacteria representative CAG-495. Mmu-miR-194-5p, along with mmu-miR-6238 and mmu-miR-6239, showed the most significant correlations in the opposite direction for the same bacterial taxa. A strong negative correlation was observed between mmu-miR-5126 and representatives of Oscillospiraceae (Lawsonibacter, Flavonifractor plautii, and Oscillibacter), Ruminococcaceae (Anaerotruncus), and Lachnospiraceae (Dorea and Acetatifactor), and Tidjanibacter (r up to -0.73, FDR < 0.001). In turn, another set represented by mmu-miR-5119, mmu-miR-192-5p, mmu-miR-191-5p, and mmu-miR-215-5p was positively correlated (r up to 0.85, FDR < 0.001) with these taxa.

#### Correlation of gut mucosal miRNAs with the gut microbiome

To assess the potential differences in the correlation pattern between various intestinal sites and to expand the range of identified potential correlations, we performed a correlation analysis using miRNAs isolated from gut mucosa samples collected from the proximal small intestine and the cecum and 16S rRNA gene sequencing data obtained both from the mucosal and the luminal microbiome of the respective sites.

**Fig 6:**
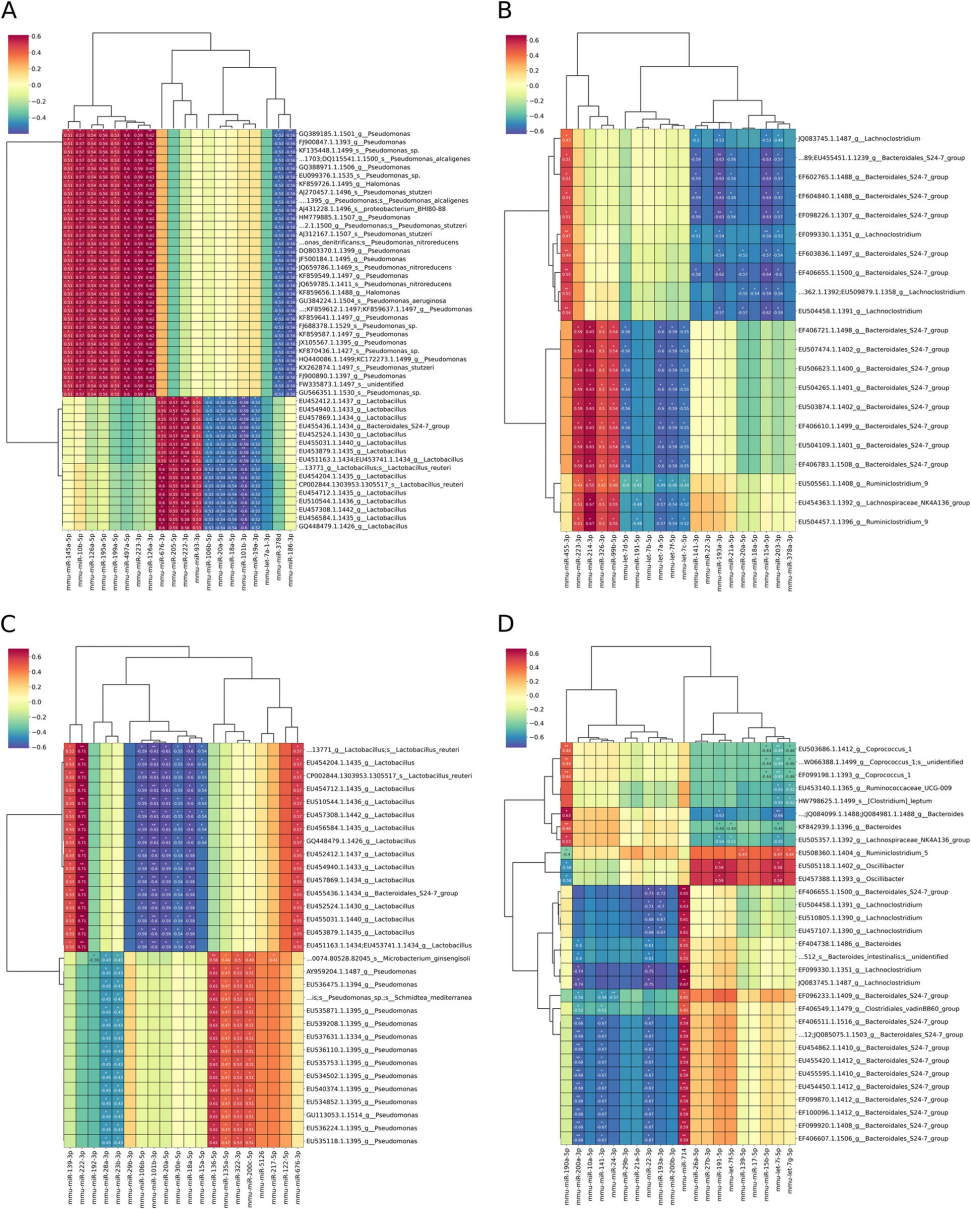
Clustered image map indicating Pearson correlation through sPLS latent components between mucosal miRNAs and gut microbiome representatives of the mucosa or lumen of the proximal small intestine or cecum identified by small RNA-seq and 16S rRNA gene sequencing, respectively. (A) Analysis between mucosal miRNAs and gut microbiome representatives of the mucosa of the proximal small intestine, N = 23. (B) Analysis between mucosal miRNAs and gut microbiome representatives of the mucosa of the cecum, N = 22. (C) Analysis between mucosal miRNAs and gut microbiome representatives of the lumen of the proximal small intestine, N = 21. (D) Analysis between mucosal miRNAs and gut microbiome representatives of the lumen of the cecum, N = 20. The color key indicates the correlation levels, with blue and red color denoting negative and positive correlation, respectively. All indicated Ns are intersections of the respective miRNA and 16S rRNA gene sequencing datasets. Only the top 20 strongest representatives from the miRNA dataset have been included in the plot. Since the analysis algorithm for the 16S rRNA gene sequencing data can assign certain features to multiple genera with the same likelihood, these features are displayed as having identical correlation levels with specific miRNAs. The heatmap is annotated with statistically significant correlation values, marked with * for FDR < 0.05 and ** for FDR < 0.001.

In the analysis subset with the mucosal microbiome (Fig 6A), the strongest negative correlation was observed between mmu-miR-101b-3p (r = -0.59, FDR < 0.001) and mmu-miR-18a-5p (r = -0.52, FDR < 0.05), among others, and the abundance of Lactobacillus and Bacteroidales S24-7 group. In turn, mmu-miR-676-3p (r = 0.6, FDR < 0.05), mmu-miR-205-5p (r = 0.57, FDR < 0.05), and mmu-miR-222-3p (r = 0.58, FDR < 0.001) showed a positive correlation with Lactobacillus. Pseudomonas was another genus, the abundance of which was strongly correlated with different sets of miRNAs. A set containing mmu-miR-10b-5p (r = 0.57, FDR < 0.05), mmu-miR-126a-3p (r = 0.62, FDR < 0.001), and mmu-miR-497a-5p (r = 0.6, FDR < 0.05) as the strongest representatives correlated positively, while a set containing mmu-miR-186-3p (r = -0.56, FDR < 0.001) as the strongest hit correlated negatively with the abundance of Pseudomonas.

In the analysis subset with the luminal microbiome (Fig 6C), the same genera were represented among the correlated ones in addition to Microbacterium. The strongest positive correlation was observed between mmu-miR-222-3p (r = 0.71, FDR < 0.001) and the abundance of Lactobacillus and Bacteroidales S24-7 group representatives and between mmu-miR-136-5p and Pseudomonas (r = 0.61, FDR < 0.05). In turn, the strongest negative correlation was detected between a set of miRNAs containing mmu-miR-101b-3p (r = -0.61, FDR < 0.001) and mmu-miR-20a-5p (r = -0.61, FDR < 0.05) and the abundance of Lactobacillus.

Analysis of samples collected from the cecum showed a distinct pattern when correlating miRNAs with mucosal or luminal microbiome members. In the mucosal microbiome analysis subset, Bacteroidales S24-7 group members showed the strongest correlation with different sets of miRNAs (Fig 6B). Two distinct sets of miRNAs were identified: one including let-7 family members (r up to -0.6, FDR < 0.05) and another set containing mmu-miR-15a-5p (r = -0.64, FDR < 0.05) and mmu-miR-193a-3p (r = -0.63, FDR < 0.001) as the strongest representatives, both negatively correlating with Bacteroidales S24-7 group. In turn, miRNAs, including mmu-miR-223-3p (r = 0.59, FDR < 0.05) and mmu-miR-214-3p (r = 0.63, FDR < 0.05), were positively correlated with the genus. Other bacteria that showed a correlation with miRNAs in this analysis subset included Ruminiclostridium 9 and Lachnospriraceae_NK4A136 group (r up to 0.67, FDR < 0.05).

The luminal microbiome analysis subset showed higher diversity of the correlated bacterial genera (Fig 6D). Similar to the analysis with the mucosal microbiome, the strongest correlation was observed between different sets of miRNAs and Bacteroidales S24-7 group. A set of miRNAs including members of miR-8 family (mmu-miR-200a-3p, mmu-miR-200b-3p, mmu-miR-141-3p), mmu-miR-10a-5p, mmu-miR-21a-5p, and mmu-miR-22-3p, among others showed a negative correlation with Bacteroidales S24-7 group (r up to -0.73, FDR < 0.05) and Lachnoclostridium (r up to -0.75, FDR < 0.05). In turn, mmu-miR-714 (r up to 0.67, FDR < 0.001) was strongly positively correlated with these genera. Another set, including let-7 family members, among others, correlated positively with Oscillibacter (r up to 0.59, FDR < 0.05) and negatively with Coprococcus_1 (r up to -0.49, FDR < 0.001) and Lachnospiraceae_NK4A136 group (r up to -0.55, FDR < 0.05) members.

### Potential bacterial targets of the identified fecal miRNAs

To investigate the potential of the identified miRNAs to biologically target the respective bacteria, the homology of all identified fecal miRNA sequences with the sequenced metagenome data was analyzed. Only the miRNAs with at least ten reads in at least 10% of the samples were included in the analysis. Furthermore, the thermodynamic stability of the miRNA-mRNA duplex, expressed as the free energy of binding between the miRNA and the target site, was evaluated. The targets of miRNAs corresponding to all of the selection criteria were denoted as hits. Complete list of bacterial species with miRNA hits is summarized in S3 Table.

**Fig 7:**
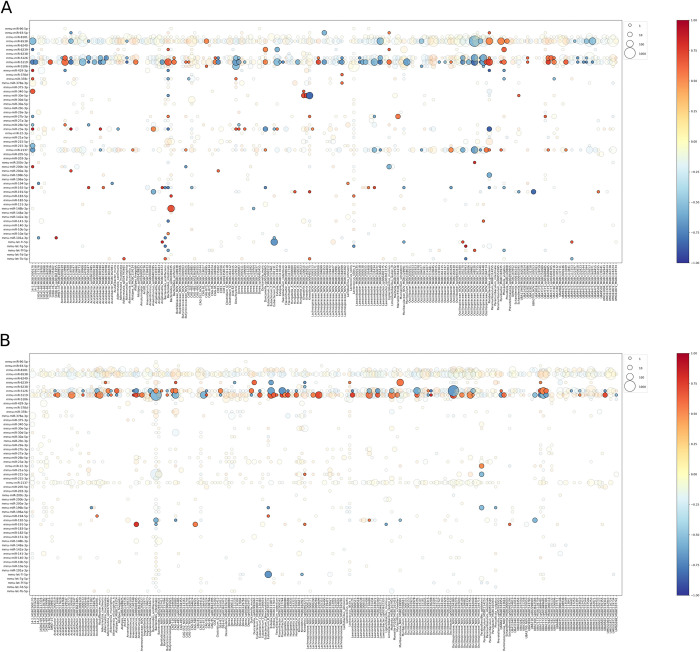
List of fecal miRNAs that have hits within bacterial genomes and correlation with the abundance of the respective bacterial species. (A) Analysis in high-fat diet-fed mice, n = 6 for correlation; n = 14 for hits. (B) Analysis in control diet-fed mice, n = 11 for correlation; n = 18 for hits. Dot sizes indicate the number of hits detected for the miRNA and bacterial species pair. The color key indicates the correlation levels, with blue and red denoting negative and positive correlations, respectively. The dot size key denotes the total number of hits detected for the miRNA and bacterial species pair. For better readability, dots with correlation levels ≤ -0.5 and ≥ 0.5 have been highlighted.

Homology assessment allowing a maximum of three mismatches revealed a median value of 7816.5 (IQR 4217.0) miRNA-read hits per sample (reads from each pair counted independently). This set of pairs was further tested based on the minimum free energy (MFE), setting a threshold of MFE values ≤ -20.0 kcal/mol. In total, this analysis retrieved 56 miRNAs and 486 bacterial species affiliated to 135 microbial genera, between which at least one potential interaction exists. Fig 7 shows the list of all potentially targeted bacterial species with at least ten hits for at least one miRNA.

As notable differences in correlation patterns were detected between experimental groups receiving different diet types, hits together with their correlation were illustrated for each dataset separately.

Homology and minimum free energy value assessment revealed that for mmu-miR-6538, at least 198 potential targets exist with at least ten total hits between samples, with the largest number of hits against Oscillospiraceae_NOV, UBA9475 (Oscillospiraceae member), and Oscillibacter. Other Oscillospiraceae members include Lawsonibacter, UMGS1872, Evtepia, and Flavonifractor. Lachnospiraceae was represented by Acetatifactor, Kineothrix, and Lachnospiraceae_NOV, while Angelakisella is affiliated with Ruminococcaceae. Bacteroidales was represented by members of five families–Muribaculaceae (UBA7173, CAG-485, Duncaniella), Prevotellaceae (CAG-873), Tannerellaceae (Parabacteroides), and Rikenellaceae (Alistipes). In turn, phylum Actinobacteriota was represented by Adlercreutzia. Bacteroides is potentially targeted by mmu-miR-5119 and mmu-miR-215-5p. Meanwhile, Lactococcus was highly targeted by mmu-miR-5106. Other top miRNAs with bacterial targets include mmu-miR-5126, mmu-miR-2137, and mmu-miR-8101.

In the HFD-fed mice subset, a stronger correlation between miRNAs and their targets was observed (Fig 7A) than in CD-fed mice (Fig 7B). Corresponding to the hit analysis, the strongest correlation was observed for mmu-miR-5119, mmu-miR-5126, mmu-miR-6538, and mmu-miR-2137. Bacterial species that are potentially targeted by the highest number of miRNAs include Bacteroides representatives with Bacteroides intestinalis as the top species, Parabacteroides goldsteinii, and 14–2 (Lachnospiraceae). Strong target potential was observed for mmu-miR-30e-5p, which is strongly negatively associated with the abundance of Kineothrix_sp000403275. Parabacteroides goldsteinii was positively correlated with mmu-miR-6538 and mmu-miR-5119, while a strong negative correlation was detected with mmu-miR-23a-3p. Oscillospiraceae_NOV members were negatively correlated with mmu-miR-6538, mmu-miR-5126, mmu-miR-5119, and mmu-miR-2137. Similar results were detected for Acetatifactor, Acutaliacter, Lawsonibacter, and UBA9475.

In the CD-fed mice subset, miRNAs that potentially target the highest number of species included mmu-miR-5126, mmu-miR-5119, mmu-miR-6239, and mmu-miR-191-5p. Bacterial species with the highest number of hits included Bacteroides intestinalis, Eubacterium_R, and Parabacteroides goldsteinii. The strongest miRNA target potential was detected for mmu-miR-5119, showing a large number of hits and negative correlation with Bacteroides intestinalis and Muribaculaceae representatives, including CAG-485, CAG-873, and UBA7173. The strongest potential targets with positive correlation include Acutalibacter, Anaerotruncus, Evtepia, Hungatella_A, Lachnospiraceae_NOV, Lawsonibacter, Oscillospiraceae_NOV, and UBA9502. A contrary potential effect was observed for mmu-miR-5126, which correlated with all of the above taxa in the opposite direction. A similar pattern was observed for mmu-miR-6239, though it targeted only a part of the targets. Another strong candidate was Eubacterium_R, which was negatively correlated with mmu-let-7i-5p.

### Functions of the identified fecal miRNAs in the host

To explore the potential host functions of the 56 identified miRNAs with bacterial genome hits, we analyzed the KEGG biological pathways involving the genes targeted by these miRNAs. The top 30 pathways, ranked by FDR values, are presented in Table 1, with a full list available in S4 Table.

**Table 1:** Host pathway analysis of fecal miRNAs that have hits within bacterial genomes. Analysis was performed using DIANA-miRPath v4.0, with predicted miRNA targets derived from TarBase v8.0 and Kyoto Encyclopedia of genes and genomes (KEGG) biological pathways. The top 30 pathways based on FDR values are shown.

KEGG pathway	Pathway genes (n)	Target genes (n)	miRNAs (n)	FDR
MicroRNAs in cancer	164	122	42	3.91E-18
Protein processing in endoplasmic reticulum	172	123	37	3.28E-016
Proteoglycans in cancer	207	133	35	7.48E-12
Renal cell carcinoma	69	57	32	7.48E-12
Ubiquitin mediated proteolysis	145	99	32	3.24E-11
Axon guidance	182	117	34	1.24E-10
Metabolic pathways	1591	741	38	1.24E-10
Salmonella infection	253	151	34	3.59E-10
Autophagy—animal	144	96	33	3.59E-10
FoxO signaling pathway	136	91	34	8.25E-10
Pathways in cancer	555	290	37	8.35E-10
Cell cycle	125	85	31	8.55E-10
Chronic myeloid leukemia	77	58	31	1.85E-09
Endocytosis	267	155	36	2.28E-09
Colorectal cancer	90	65	34	2.60E-09
Pancreatic cancer	78	58	34	3.64E-09
AMPK signaling pathway	130	86	35	4.23E-09
T cell receptor signaling pathway	106	73	35	5.77E-09
Hepatitis B	166	104	38	6.13E-09
Longevity regulating pathway	92	65	33	9.02E-09
Pathways of neurodegeneration—multiple diseases	480	251	36	9.52E-09
Adherens junction	71	53	33	1.29E-08
AGE-RAGE signaling pathway in diabetic complications	103	70	36	2.48E-08
Thyroid hormone signaling pathway	122	80	35	2.48E-08
Human T-cell leukemia virus 1 infection	244	140	35	3.11E-08
Insulin signaling pathway	143	90	35	4.98E-08
Focal adhesion	205	120	35	8.13E-08
Insulin resistance	115	75	33	9.60E-08
Neurotrophin signaling pathway	123	79	33	1.05E-07
ErbB signaling pathway	87	60	34	1.22E-07

Of the analyzed miRNAs with hits in bacterial genomes, no targets in the host were found for mmu-miR-2137, mmu-miR-215-3p, mmu-miR-5106, mmu-miR-5119, mmu-miR-5126, mmu-miR-6238, mmu-miR-6239, mmu-miR-6240, mmu-miR-378c, mmu-miR-378d, mmu-miR-6538, and mmu-miR-8101 with the selected resources. The top pathways identified include MicroRNAs in cancer and Protein processing in endoplasmic reticulum. Other significantly enriched pathways, relevant to the context of diabetes, include Metabolic pathways, FoxO signaling pathway, AMPK signaling pathway, Insulin signaling pathway, and Insulin resistance pathway, among others.

### Proteins targeted by the identified fecal miRNAs and their functions

**Fig 8:**
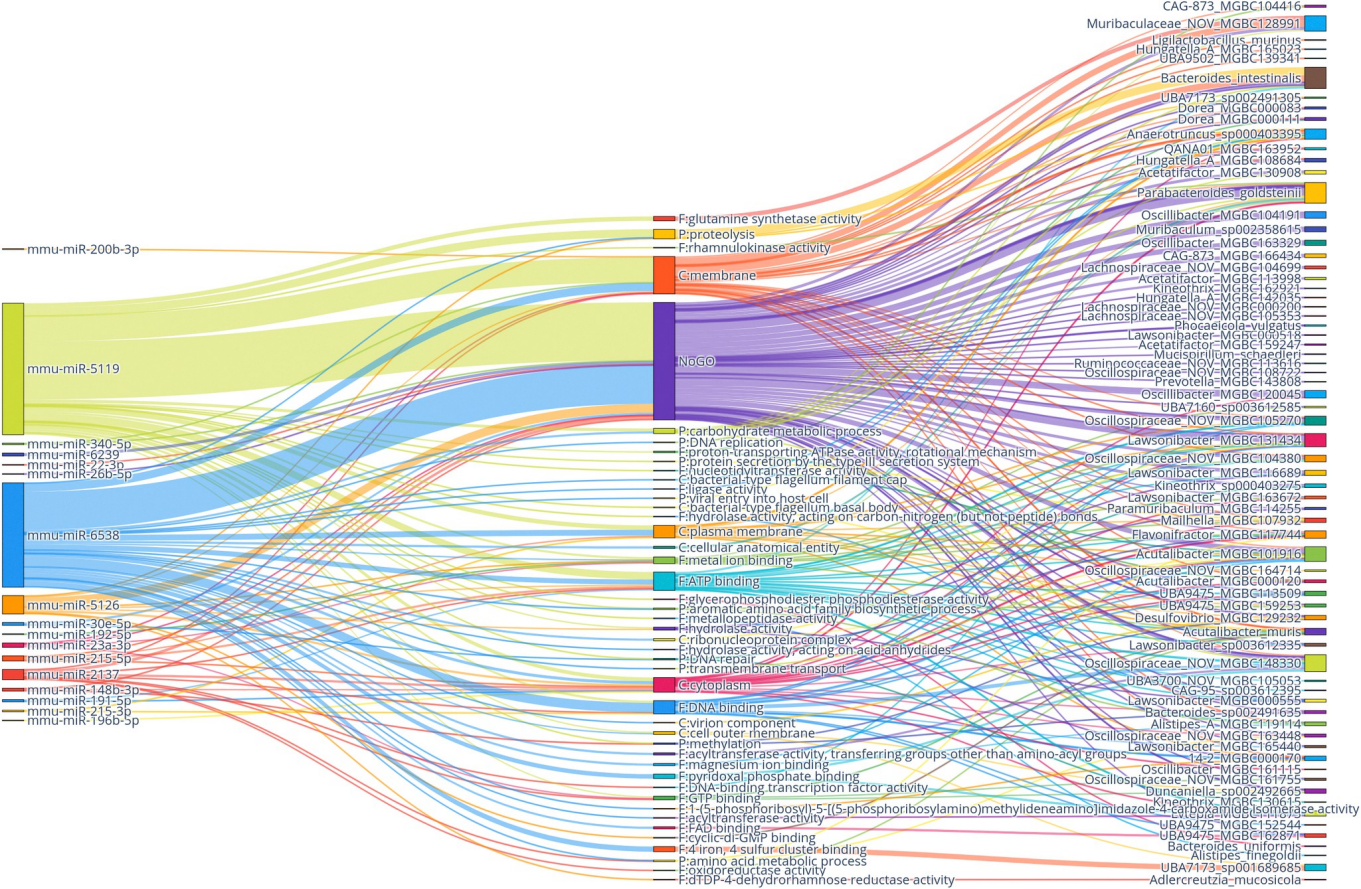
A sankey plot illustrating the interaction between fecal miRNAs, their bacterial targets and targeted proteins within these bacteria. Only miRNAs and bacteria with the following combinations of number of hits and correlation are included in the plot: ≥ 100 hits and correlation ≥ 0.4; ≥ 10 hits and correlation ≥ 0.5; and ≥ 1 hit and correlation ≥ 0.8. Proteins have been grouped by their gene ontology (GO) term: F–molecular function; P–biological process; C–cellular component. Proteins without corresponding GO term have been combined together and denoted as NoGO.

To further analyze the mechanisms and networks that are involved in the bacterial regulation by host miRNAs, we performed an analysis in which target sequences were translated into proteins, and their functions were investigated for each bacterial species separately. The analysis yielded a list of miRNA/targeted protein/bacterial species triples in which a total of > 25000 unique triples were identified. The complete list of triples has been summarized in S5 Table. Triples with the highest hit counts and the strongest correlation were summarized in a plot shown in Fig 8.

Triple analysis revealed that targeted proteins can be affiliated with cellular components, including the plasma membrane, cytoplasm, cell outer membrane, ribonucleoprotein complex, virion component, and bacterial-type flagellum filament cap and basal body. Biological processes include proteolysis, DNA replication, methylation, amino acid metabolic process, carbohydrate metabolic process, viral entry into host cell, DNA repair, and transmembrane transport, among others. Molecular functions comprise a list of different binding functions–DNA, ATP, GTP, FAD, magnesium ion, metal ion, pyridoxal phosphate, and cyclic-diGMP binding. Other functions include the activity of proton-transporting ATPase, rotational mechanism; ligase; hydrolase; oxidoreductase; metallopeptidase; rhamnulokinase, and acyltransferase, among others.

Triple analysis revealed that most of the targeted proteins are related to efflux functions and transmembrane transport (Fig 8). The highest number of hits were identified for Bacteroides (mainly B. intestinalis) representatives and the mmu-miR-5119 or mmu-miR-215-5p. Mmu-miR-5119 potentially targets ABC transporter-related proteins and dipeptidyl-peptidases. Potential targets for mmu-miR-215-5p in Bacteroides include ATPase domain-containing proteins and DUF4980 domain-containing protein. STRING analysis of functional enrichments in the network using proteins targeted by mmu-miR-5119 in Bacteroides intestinalis revealed significant enrichment for ATP-binding cassette (ABC) transporter complex (strength = 2.17, FDR = 0.007) and Membrane protein complex (strength = 1.55, FDR = 0.007). In turn, for mmu-miR-148b-3p, the main target is serine hydroxymethyltransferase. Another top miRNA with bacterial targets mmu-miR-6538, mainly targeted various transporter proteins in Acetatifactor and Acutalibacter and a wide variety of proteins in Oscillospiraceae members (Lawsonibacter, Oscillibacter, Oscillospiraceae_NOV, and UBA9475), among others. STRING analysis using proteins targeted by mmu-miR-6538 in Oscillospiraceae_NOV members identified significant enrichment in KEGG pathway RNA degradation (strength = 1.53, FDR = 0.01).

Differences in protein targets by various miRNAs targeting the same bacterial genus indicate their specificity. According to our analysis, serine hydroxymethyltransferase is uniquely targeted by several miRNAs: mmu-miR-148b-3p (Bacteroides spp.), mmu-miR-6538 (Acutalibacter, UBA11940, and Oscillospiraceae members including UBA9475), and mmu-miR-5126 (Kineothrix). Furthermore, in different species from the same genus, different proteins can be targeted by the same miRNA. For example, mmu-miR-148b-3p targets only LPS-assembly protein LptD in Bacteroides intestinalis, while in Bacteroides uniformis, this miRNA exclusively targets serine hydroxymethyltransferase.

**Fig 9:**
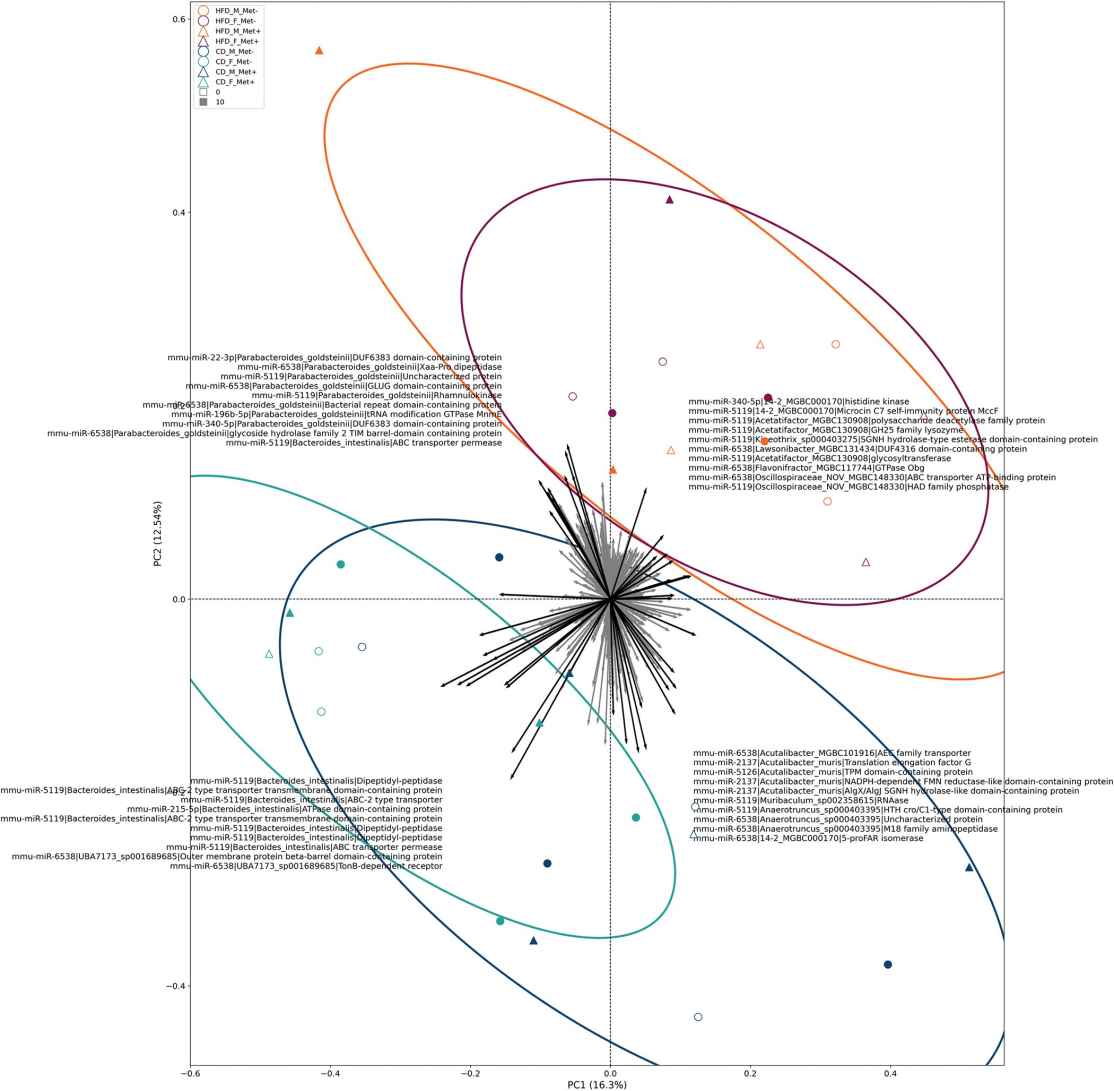
A biplot illustrating the principal identifiers composed as miRNA, protein, and bacterial target trios of each experimental group in fecal samples. N = 32.

Analysis was continued by investigating whether triples differ between experimental groups, thus potentially indicating the effect of studied factors on the interaction between miRNAs and bacteria (Fig 9). A strong division depending on diet type was detected, and to a lesser extent, sex-dependent effects were observed. HFD-fed groups were characterized by the interaction between Parabacteroides goldsteinii and mmu-miR-22-3p and mmu-miR-340-5p (both targeting DUF6383 domain-containing protein), mmu-miR-6538, mmu-miR-5119, and mmu-miR-196b-5p. In addition, mmu-miR-5119 targeted Acetatifactor, Kineothrix, and Lachnospiraceae representative 14–2, which was also targeted by mmu-miR-340-5p with histidine kinase as a protein target. Mmu-miR-6538 targeted Lawsonibacter and Flavonifractor, while both mmu-miR-6538 and mmu-miR-5119 targeted Oscillospiraceae_NOV.

In turn, CD-fed experimental groups were characterized by the interaction between mmu-miR-2137 or mmu-miR-6538 and Acutalibacter; mmu-miR-5119 and Muribaculum; and mmu-miR-5119 or mmu-miR-6538 and Anaerotruncus in one axis and mmu-miR-5119 or mmu-miR-215-5p and Bacteroides intestinalis; and mmu-miR-6538 and Muribaculaceae member UBA7173 in another. Proteins targeted in Bacteroides intestinalis included dipeptidyl-peptidase and ABC transporter-related proteins. Outer membrane-related proteins were targeted in UBA7173. The interacting miRNAs were generally shared between all of the principal directions of the biplot, together with some axis-specific miRNAs.

## Discussion

Encouraged by the findings of Liu and colleagues [3]⁠, we hypothesized that host miRNAs are mediators in metformin-induced changes in the gut microbiome composition and functions in the context of type 2 diabetes. To explore this hypothesis, we investigated the effect of ten weeks long metformin treatment on miRNA expression in fecal and gut mucosal samples representing proximal small intestine and cecum of diabetic and control group mice. We then combined miRNA datasets with respective microbiome composition datasets, which were obtained and described in previous studies using the same animals [28, 29]. To our best knowledge, this is the first study to address the effect of metformin on fecal and intestinal miRNA composition and its implications on gut microbiome.

Our results show that the repertoire of miRNAs in fecal samples is relatively consistent despite variations in time and treatment. The fact that at least 20 miRNAs were represented in each of the experimental groups, including longitudinal samples, provides information on the overall stability of the fecal miRNA content over the time, marking them as consistent players of the microbiome ecosystem.

All miRNAs from the top 20 list have been shown previously to be present in fecal samples of mice and humans [3, 5, 11, 13, 22, 30–32]⁠. It should be noted that we have used a sequencing-based approach compared to many of the other studies, thus providing an unbiased view of fecal miRNA content. Taken together, regarding the top miRNAs, the gut mucosal miRNA composition of the proximal small intestine was similar to that of the cecum; almost all top 20 miRNAs corresponded between sites, though the relative abundances of miRNAs were different.

Differential expression analysis between the studied groups mainly revealed diet-related, intestinal part-dependent, and some sex-related effects on miRNA composition, while metformin had a significant effect only in one of the examined contrasts. MiRNAs enriched in the proximal small intestine included both strands of each–mmu-miR-802, mmu-miR-31, and mmu-miR-215, whereas mmu-miR-8101, mmu-miR-196a-5p, mmu-miR-196b-5p, mmu-miR-5100, and mmu-miR-5119 were increased in the cecum.

We observed a minor effect of diet type on miRNA expression in gut mucosal samples, whereas the effect was substantial in fecal samples. Dietary lipid effects on intestinal miRNAs have been characterized previously and have shown a large number of miRNAs that are influenced in response to high-fat diet feeding [33]. A number of the miRNAs increased in the proximal small intestine, including miR-215 members, miR-141 members, and mmu-miR-22-3p, are shown to be enriched in the Sox9Neg cells (enterocytes, goblet cells, and Paneth cells) [34].

A recent study has demonstrated estrogen-mediated protection against insulin resistance in females through the miR-10a/b-5p-NCOR2 axis, both in mice and humans [35]. Another study has reported a significant interaction between sex and study groups with various glycemic statuses in the expression changes of miR-10a-5p within the context of diabetes [36]. These findings align with our observations of increased expression of mmu-miR-10a-5p in HFD-fed female mice compared to males, which corresponds with the lower insulin resistance observed in the female mice, as reported in our previous study [28].

In our study, we observed a limited impact of metformin treatment on miRNA expression, which may be attributed to several factors. MiRNA regulation is a complex process influenced by tissue specificity, cellular context, and drug dosage. Previous research has demonstrated that metformin’s effects on gene expression and miRNA levels can vary across different cell types and animal models [37]. Metformin is primarily absorbed in the small intestine, with higher concentrations found in the proximal regions [27], potentially leading to localized changes in gene expression, including miRNAs, where the drug concentration is highest. Additionally, we administered a relatively low dose of metformin (50 mg/kg/day), which is considered to be the maximum dose that the body can efficiently utilize [38]. Therefore, while metformin had modest effects on miRNA profiles in our study, these findings underscore the need for further research into the nuanced interactions between therapeutic agents, miRNA regulation, and metabolic diseases such as diabetes and insulin resistance.

Despite the lack of substantial effect of metformin treatment on miRNA expression, we investigated the interaction between host miRNAs and members of the gut microbiome depending on the type of dietary intake. The first step was to assess the correlation pattern between host miRNAs and bacterial species. Correlation analysis showed the ability of certain miRNAs to distinguish bacteria at the family and genus levels. Thus, specific groups of miRNAs were inversely associated with the Akkermansia and the genera from Lachnospiraceae (with some exceptions), Oscillospiraceae, and the genera from Bacteroidaceae and Muribaculaceae (both from Bacteroidales). Most of the miRNAs grouped based on their affiliation within miRNA families. Strong example of this is miR-8 family with its members–mmu-miR-200a-3p, mmu-miR-200b-3p, mmu-miR-200c-3p, mmu-miR-141-3p, and mmu-miR-429-3p.

Further bioinformatic analysis was conducted to explore the capability of the correlated miRNAs to biologically target the corresponding bacteria and to reinforce the findings of the correlation analysis. Only datasets of fecal sample analysis were used for further investigation due to possibility to directly map miRNA sequences against metagenomic sequencing reads.

When analysis of potential bacterial targets in feces was combined with respective correlation analysis, several miRNA and bacterial species pairs were identified. Both mmu-miR-5126 and mmu-miR-5119 potentially target several Oscillospiraceae species and Muribaculaceae representative CAG-873 and affect their abundance in opposite directions. This is consistent with the results of the fecal metagenome analysis, where we have shown a lower relative abundance of Bacteroidales, including Muribaculaceae members and an increase in Oscillibacter in the feces of high-fat diet-fed mice compared to control diet-fed ones both of which were altered by metformin treatment [28].

Oscillospiraceae and Muribaculaceae are key families of gut bacteria involved in fermenting dietary fibers and mucin glycans, playing crucial roles in maintaining gut health and regulating metabolic processes [39, 40]. Muribaculaceae, in particular, are known for their role in digesting complex carbohydrates such as mucin glycans and producing acetate and propionate. These short-chain fatty acids (SCFAs) help to maintain gut barrier integrity, modulate the immune response, and promote overall metabolic health [40].

Oscillibacter species, recognized as butyrate producers, have been associated with lower fecal and plasma cholesterol levels in the context of cardiovascular disease [41]. However, in HFD-fed mice, the abundance of Oscillibacter has been shown to increase, with positive correlations observed between its abundance and elevated body weight, glucose, and cholesterol levels [42].

Overall, our analysis highlighted a list of miRNAs that are correlated with their bacterial targets and have protein targets in these bacteria. Furthermore, many of these miRNAs were identified to be differentially expressed depending on various host-related factors. Among these miRNAs, mmu-miR-5119, mmu-miR-5126, mmu-miR-8101, mmu-miR-6538, and mmu-miR-2137 especially stood out. According to Tomkovich et al., fecal miRNAs primarily targeting bacterial genes can be distinguished separately from those targeting the host’s genes. In their study on the interaction between colon mucosal biofilms and mice hosts after the host inoculation with colorectal cancer patient-derived microorganisms, several miRNAs that mainly target bacterial genes have been identified [5]. Interestingly, their list of bacteria-targeting miRNAs fully corresponds to our top miRNAs having bacterial targets. Furthermore, our functional analysis of these miRNAs did not detect any host gene targets, underscoring their distinct specificity for bacterial interactions. The fact that we observed a significant increase of these miRNAs in the cecum compared to the proximal small intestine further strengthens their potential role in targeting gut microbiome, as bacterial diversity and density increases longitudinally along the gastrointestinal tract [43]. The distribution of miRNAs has been reported to have the opposite pattern, with miRNAs being more abundant in the ileal lumen in contrast to the colon [3]. It could be speculated that the types of miRNAs involved in targeting bacteria depend on their distribution in the gastrointestinal tract and may be related to the distribution of the intestinal cell types that secrete them throughout the gastrointestinal tract. Previous research has suggested that gut epithelial cells and Hopx-expressing cells (Paneth cells and goblet cells), the two main sources of fecal miRNAs, produce distinct sets of miRNAs [3].

Interestingly, in accordance with previous research [5], our results indicate that bacteria-targeting host miRNAs are typically identified by higher numerical values in their nomenclature, indicating they are relatively recent discoveries. It should be noted, that a high number in the name of the miRNA is not an absolute requirement for it to potentially target bacteria as miR-30d-5p has been experimentally shown to upregulate the expression of β-galactosidase in Akkermansia muciniphila in experimental autoimmune encephalomyelitis mouse model of multiple sclerosis [11]. In another study, miR-21 was shown to directly target Lactobacillus growth in the background of liver dysfunction [9]. This indicates that relatively well-described and common miRNAs are also potentially involved in regulating bacterial growth. Our results have shown that miR-215-3p, miR-215-5p, miR-22-3p, miR-141-3p, and miR-194-5p, among others, are examples of such.

Finally, we assessed which proteins are potentially targeted by each of the previously identified miRNAs in each of their bacterial targets. Intestinal bacteria require a variety of proteins for their growth and survival in the complex environment of the gastrointestinal tract. Some essential proteins for the growth of intestinal bacteria include: enzymes involved in carbohydrate metabolism; transport proteins; enzymes involved in amino acid metabolism; cell wall synthesis enzymes; ribosomal proteins and translation machinery; metabolic enzymes involved in energy production; and stress response proteins [44–47]. Our triple analysis revealed that all of these protein types are represented among those targeted by host miRNAs. Frequently found proteins in our results include ABC transporters, ATP-binding proteins, metalloproteinases, and various membrane-related proteins. Cellular components included bacterial-type flagellum filament cap and basal body, as well as virion component and viral entry into host cell.

The top targeted proteins by mmu-miR-5119 were ABC transporters and dipeptidyl-peptidases which, in agreement with our results, previously have been reported to be targeted by mmu-miR-6538, mmu-miR-5119, mmu-miR-6236 and several other miRNAs [5]. In contrast, we observed mmu-miR-6538 and mmu-miR-148b-specific targeting of serine hydroxymethyltransferase, which was targeted only by mmu-miR-2137 in previous research [5].

While some miRNAs may exhibit broad effects on multiple bacterial species or strains, others may show more specific interactions with particular bacterial species. Our analysis showed considerable specificity of miRNAs as, in several instances, one protein is targeted by one miRNA in one bacterial species. However, the same miRNA can target different proteins in different species from the same genus. Understanding the determinants of miRNA specificity and selectivity for bacterial targets is important for elucidating their roles in host-gut microbiome interactions.

Overall, while the concept of host miRNAs targeting bacteria is still relatively new, accumulating evidence suggests that miRNAs play important roles in host-microbiome interactions and may represent novel targets for therapeutic intervention in microbiome-related conditions. Our results, together with previous studies in different conditions, including multiple sclerosis, inflammatory bowel diseases, and colorectal cancer [11, 31, 32], support the notion that host miRNAs in fecal samples may mediate changes in gut microbiome composition by targeting specific bacteria and their protein-encoding genes. While our study contributes to this growing body of evidence, further research is needed to fully elucidate the mechanisms and significance of miRNA-mediated regulation of bacteria. Functional validation studies are essential to confirm the observed correlations and their impact on microbiome dynamics and host health. Nevertheless, these findings open new avenues for hypothesis generation and potential therapeutic applications, highlighting the intricate interplay between host miRNAs and the gut microbiome in health and disease.

A limitation of our study is the relatively low fecal miRNA sequencing depth, and the results would benefit from analysis in larger groups. While we did not detect a significant metformin’s effect on miRNA expression, it is possible that such effects exist in less expressed miRNAs that were not reached by the current sequencing depth in fecal samples. However, since no consistent effect was observed in mucosal samples as well, which had substantially higher sequencing depth, this suggests that metformin may not have a significant impact on miRNA expression in this context. Functional studies are recommended to validate these findings further.

In conclusion, our study represents the first investigation into the relationship between host fecal miRNAs and gut microbiome composition in the context of type 2 diabetes. Although we did not find consistent significant differences in miRNA expression in response to metformin treatment, our findings revealed significant variations in miRNA expression based on diet type, modeling type 2 diabetes status, and between different intestinal parts, as well as some sex-related differences.

Our study showed that host miRNAs can target bacteria in fecal samples, reinforcing the idea that the host is able to modulate its microbiome via its miRNAs. Diabetes background had a strong effect on the extent of the interaction between host miRNAs and their bacterial targets and, in some cases, even reversing the correlation direction. Top bacteria-targeting fecal miRNAs include mmu-miR-5119, mmu-miR-5126, mmu-miR-6538, and mmu-miR-2137, with primary bacterial targets such as Oscillospiraceae_NOV, Lachnospiraceae_NOV members, and Bacteroides representatives. Proteins targeted by host miRNAs mainly included various transporters and membrane-related proteins, indicating changes in transmembrane transport and efflux functions essential for bacterial survival and growth.

Our findings highlight the intricate interplay between host miRNAs and the gut microbiome in type 2 diabetes and emphasize the importance of considering disease background and other factors in microbiome studies. Future research should focus on elucidating the functional significance of miRNA-mediated regulation of bacteria and exploring potential therapeutic applications in microbiome-related conditions.

## Materials and methods

### Ethical approval

Animal procedures were reviewed and approved by the National animal welfare and ethics committee (Permit No. 91) and performed in compliance with Directive 2010/63/EU as adopted in the national legislation.

### Animals and study design

The animal experiment has been described previously in [28]. In brief, C57BL/6N mice of both sexes with specific pathogen-free status (SPF) were purchased from the University of Tartu Laboratory Animal Centre and acclimatized to the local animal facility for one week. At the beginning of the study, all mice were six weeks old. All mice were housed under SPF conditions at a temperature of 23 ± 2°C and 55% humidity, with a 12:12-hour light cycle (light period from 7:00 am to 7:00 pm). Animals were housed in individually-ventilated cages (Tecniplast) with up to three same-sex animals per cage, furnished with aspen bedding mixed with ALPHA-dri. Depending on their experimental group affiliation, which was randomly assigned at the beginning of the study, the mice were provided ad libitum access to either a high-fat diet (HFD) (rodent diet with 60 kcal% fat (D12492, Research Diets)) or a control diet (CD) (rodent diet with 10 kcal% fat (D12450J, Research Diets)), along with free access to drinking water. A total of 72 animals were included in the study, forming 24 experimental units (cages with animals). According to the resource equation method, appropriate for complex designs [48]⁠⁠, the study’s sample size was adequate. Throughout the entire experiment, animals underwent daily observation. In instances where any form of suffering was observed that could not be relieved, the animal was euthanized by cervical dislocation. A humane endpoint was implemented for 13 animals during the study, primarily due to injuries sustained from male fighting. The experimental groups represented by the excluded animals are summarized in the S6 Table. In sequencing analysis, each experimental unit was represented by one animal from the corresponding cage.

The experimental design of the present study has been illustrated in Fig 1. The total duration of the study was 30 weeks (excluding the adaptation period), of which 20 weeks were for the induction of T2D manifestations by HFD feeding and 10 weeks were for metformin treatment provided with drinking water (50 mg/kg body mass/day). At the termination of the study, all the animals were sacrificed by cervical dislocation.

The study had a randomized block design. The study involved eight different treatment groups designated as HFD_M_Met-, HFD_F_Met-, HFD_M_Met+, HFD_F_Met+, CD_M_Met-, CD_F_Met-, CD_M_Met+, CD_F_Met+, depending on diet type (HFD or CD for high-fat diet or control diet, respectively), sex (M or F for males and females respectively), and metformin treatment status (Met+ or Met- for groups receiving either treatment or no treatment with metformin). Each of the eight treatment groups was represented by three experimental units (cages with animals), 24 in total. In addition to cross-sectional contrasts between experimental groups, each of them was analyzed longitudinally by using fecal samples collected at the time points before the initiation of metformin treatment, and after ten weeks long metformin treatment designated by adding _0 and _10 to the corresponding experimental groups, respectively, therefore, the total number of fecal samples was 48. Fecal samples were collected in sterile tubes filled with RNALater after the animals voluntarily defecated into clean, separate boxes where they were temporarily placed during cage changes. Intestinal mucosa samples representing the proximal small intestine (n = 24) and cecum (n = 24) were collected at the end of the experiment –after ten weeks-long oral metformin treatment. Given the variation in gut microbiome composition throughout the gastrointestinal tract, we hypothesized that host miRNA-microbiome interactions would differ across various gut locations. We expected the most pronounced differences in these interactions to occur between the proximal small intestine (the primary site of metformin absorption), the cecum, and feces. Therefore, samples from these sites were selected for miRNA analysis. Type 2 diabetes model protocol, biochemical parameters, and sample collection were described in [28, 29] in detail.

### Microbial DNA isolation and microbiome sequencing

Sample preparation, shotgun metagenomic sequencing of fecal samples, and 16S rRNA gene sequencing of intestinal mucosa samples, together with the respective data analysis methods, have been described previously in [28, 29].

### Fecal RNA isolation and small RNA sequencing

Total RNA from fecal samples, stored in RNAlater, of mice representing each experimental unit at the two time points–before and after the metformin treatment was isolated using All Prep DNA/RNA/miRNA Universal Kit (QIAGEN) according to the manufacturer’s instructions. Qubit RNA HS Assay Kit on the Qubit 2.0 (Invitrogen Co.) was used to determine the concentration of isolated RNA. The quality of the extracted RNA samples was analyzed using Agilent 2100 Bioanalyzer (Agilent, USA) and Agilent Small RNA Kit (Agilent, USA).

Before library preparation, QC Spike-ins (QIAGEN, Germany) were added to the samples. MiRNA libraries were prepared using QIAseq miRNA Library Kit (QIAGEN), with 100 ng of isolated RNA input from each sample. Library preparation steps briefly: 3’ ligation; 5’ ligation; reverse transcription, converting miRNAs into cDNA while assigning unique molecular indices (UMIs) to every miRNA molecule followed by cDNA cleanup using the magnetic bead-based method; library amplification, where a universal forward primer is paired with reverse primers to assign to each sample followed by library cleanup using the magnetic bead-based method; quality control by Qubit dsDNA HS Assay Kit on the Qubit 2.0 (Invitrogen Co.) and Agilent High Sensitivity DNA Kit (Agilent Technologies) on the Agilent 2100 Bioanalyzer (Agilent Technologies).

The prepared libraries were sequenced on MiSeq System (Illumina) using MiSeq Reagent Kit v3 for 150 cycles (Illumina), following the manufacturer’s instructions. Sequencing depth was calculated to achieve at least 6 million single-end small RNA-seq reads per sample.

### Gut mucosal RNA isolation and small RNA sequencing

Total RNA from mucosal samples, stored in RNAlater, of mice representing each experimental unit and collected from two intestinal parts–proximal small intestine and cecum was extracted using All Prep DNA/RNA/miRNA Universal Kit (QIAGEN) according to the manufacturer’s instructions. Qubit RNA HS Assay Kit on the Qubit 2.0 (Invitrogen Co.) and Agilent Small RNA Kit (Agilent, USA) on the Agilent 2100 Bioanalyzer (Agilent, USA) were used to measure the concentration of isolated RNA and to assess the quality of the extracted RNA samples, respectively.

MiRNA libraries were prepared using MGIEasy Small RNA Library Prep Kit, the input of total RNA was 100 ng per sample. Preparation of the libraries involved: 3’ ligation; 5’ ligation; reverse transcription, library amplification; cleanup with magnetic beads; quality control by Qubit dsDNA HS Assay Kit on the Qubit 2.0 (Invitrogen Co.) and Agilent High Sensitivity DNA Kit (Agilent Technologies) on the Agilent 2100 Bioanalyzer (Agilent Technologies); pooling of libraries; denaturation; circularization; enzymatic digestion and cleanup of the digestion product by magnetic beads; and quality control by Qubit ssDNA Assay Kit and Agilent High Sensitivity DNA Kit.

The prepared libraries were sequenced on DNBSEQ-G400RS sequencing platform (MGI Tech Co. Ltd) using Small RNA FCL SE50 (MGI Tech Co. Ltd) following the manufacturer’s instructions. At least 16 million 50-bp single-end reads per sample were expected to be obtained. The number of sequencing reads obtained ranged from 11.4 million to 31.7 million reads per sample.

### Data analysis

#### Small RNA sequencing data analysis

Data were analyzed using CLC Genomics Workbench 20.0.4. and Galaxy Release v21.01. For fecal samples read quality assessment was performed with QC for Sequencing Reads. Reads were trimmed using a quality score of 0,012. 3’ and 5’ adapter trimming as well as sequence filtering on length (reads below 15 nt and above 55 nt were discarded) was performed. Read quality of gut mucosal samples was evaluated by the Galaxy platform using FastQC (v0.11.8). Adapters were removed using Cutadapt (v.1.16), indicating AAGTCGGAGGCCAAGCGGTCTTAGGAAGACAA as a 3`adapter sequence and AAGTCGGATCGTAGCCATGTCGTTCTGTGAGCCAAGGAGTTG as a 5`adapter sequence. Quantify miRNA was used to map the reads against miRBase release v22, pointing out Mus musculus as prioritized species. Reads fixed for seed counting had a minimum sequence length of 18 nt and a maximum sequence length of 25 nt. Spike-in controls were enabled and reference from QIAseq Small RNA (Version 1.0) dataset was used. RNA-Seq Analysis tool was used to map the reads against mouse reference genome Ensembl v86. Reads were mapped allowing 2 mismatches.

Differential expression was tested with edgeR package 3.32.1 and limma 3.46.0 using voom transformation with sample quality weights. Differential testing was executed for combinations of multiple factors: diet type, sex, metformin treatment, and intestinal part or time. Changes in miRNA expression between experimental groups were expressed as LogFC (logarithmic fold change with base 2). Multiple testing correction was implemented using the Benjamini-Hochberg procedure. MiRNAs with a false discovery rate (FDR) ≤ 0.05 were noted as statistically significantly altered.

#### Correlation analysis

Taxa and miRNAs with at least 100 and 10 reads in 10% of samples, respectively, were retained for further analyses. Fecal samples with less than 1000 or 100 000 miRNA or classified metagenomic reads, respectively, were removed. Mucosal samples with less than 2000 miRNA or metagenomic reads were removed. Prior to centered log ratio normalization (clr), an arbitrary constant (value 1) was added to all values in order to enable clr. Correlation analysis of metagenomic read counts and miRNA read counts was performed by transforming count values with clr and subsequent sparse partial least squares regression (sPLS) as implemented in R package mixomics 6.20.0. Correlation values were visualized as clustered image maps (CIM) using the mixOmics framework. Within mixomics framework, the correlations were calculated between miRNA and latent components, as well as between metagenomic data and latent components, with the latent components estimated during the sPLS process. The dot product of both correlation matrices formed final values in CIM.

P-value calculation of CIM values was performed with the approximate permutation test. During p-value calculation miRNA labels from correlation matrix of mirna clr values with the sPLS estimated metagenomics components were shuffled and CIM matrix was recalculated 100 000 times. For each analyzed group, p-values were obtained by counting values exceeding the originally observed effect size and dividing the obtained counts by the number of iterations. Adjustment for multiple corrections was performed using the Benjamini-Hochberg procedure as implemented in SciPy.

#### Bacterial target analysis

Potential binding sites for all miRNAs that had at least 10 reads in 10% of samples in either HFD or CD groups were evaluated in the corresponding sample metagenomic sequencing data. Mature miRNA sequences were obtained from miRBase database (release 22.1). Samples with at least 1000 miRNA reads in total were considered.

Candidate bacterial targets were estimated from hybridisation potential, however, since minimum free energy calculation (MFE) is a relatively resource-intensive task, we first screened for reads that had at most three mismatches/indels with the miRNAs as a heuristic. The number of mismatches/indels was calculated using Smith-Waterman’s algorithm for sequence alignment. For candidate reads, the MFE was calculated with RNAup 2.5.1. All reads with MFE values ≤ -20.0 kcal/mol were considered hits. Taxonomic classification of hits was then performed with Kraken 2.0.8-beta against the MGBC database [49], and the number of hits per taxa per miRNA was aggregated as a table. During taxonomic classification confidence threshold value 0.1 was applied.

#### Host target analysis

Host pathway analysis of fecal miRNAs that have hits within bacterial genomes (N = 56) was performed using DIANA-miRPath v4.0 [50], using predicted miRNA targets derived from TarBase v8.0 and Kyoto Encyclopedia of genes and genomes (KEGG) biological pathways. Mus musculus was selected as target species, miRBase-v22.1 as the annotation database, and Genes union as the merging method. KEGG pathways with a false discovery rate (FDR) ≤ 0.05 were considered as statistically significantly enriched.

#### Analysis of targeted proteins in bacteria

Reads that potentially bind miRNAs (with MFE ≤ -20.0 kcal/mol) were aligned against Uniref90 (release 2023_03) sequence clusters with diamond 2.1.8 blastx. Reads with at least 90% identity and 80% query coverage were considered as potential hits. Up to 25 hits per read were considered. For each sequencing read-pair best hit was assigned as follows: intersecting hits (by uniref90 id) of both reads in a pair were identified. Best hit was selected from intersecting hits as hit with the highest bitscore. If there were multiple best hits, one of them was randomly selected as the final hit. In cases with no intersecting hits, the final hit was selected from the union of hits from both reads in a pair.

All hit types for each read-pair (Uniref90, miRNA and taxonomic classification) were joined, forming triples. Per sample aggregation of triple counts was performed, forming a count matrix for further statistical analyses. STRING database was used to assess functional enrichments in protein-protein interaction networks [51].

Data visualizations were performed with matplotlib 3.6.3, plotly 5.18.0. Principal component analysis was performed with scikit-learn 1.4.1.post1. Clustered image maps were generated from sPLS results with mixomics 6.20.0.

## Supporting information

S1 Table Differentially expressed miRNAs in fecal samples before (0) and after (10) the metformin treatment, N = 32. Comparisons in which statistically significant differences have been detected are shown. FDR–false discovery rate.
(XLS)

S2 Table Differentially expressed miRNAs in mucosal samples of proximal small intestine (PSI) or cecum (CEC). N = 45. Comparisons in which statistically significant differences have been detected are shown. FDR–false discovery rate.
(XLS)

S3 Table List of miRNAs and their bacterial targets in fecal samples. (XLS)

S4 Table Host pathway analysis of fecal miRNAs that have hits within bacterial genomes. (XLS)

S5 Table List of miRNA/targeted protein/bacterial species triples identified in fecal samples. (XLS)

S6 Table Excluded samples and animals from the analysis. (XLS)
